# Improving transient protein expression in agroinfiltrated *Nicotiana benthamiana*

**DOI:** 10.1111/nph.19894

**Published:** 2024-06-07

**Authors:** Konstantina Beritza, Emma C. Watts, Renier A. L. van der Hoorn

**Affiliations:** ^1^ The Plant Chemetics Laboratory, Department of Biology University of Oxford OX1 3RB Oxford UK

**Keywords:** *Agrobacterium tumefaciens*, agroinfiltration, ER stress, gene silencing, immunity, *Nicotiana benthamiana*, proteolysis, transient expression

## Abstract

Agroinfiltration of *Nicotiana benthamiana* is routinely used in plant science and molecular pharming to transiently express proteins of interest. Here, we discuss four phenomena that should be avoided to improve transient expression. Immune responses can be avoided by depleting immune receptors and employing pathogen‐derived effectors; transcript degradation by using silencing inhibitors or RNA interference machinery mutants; endoplasmic reticulum stress by co‐expressing chaperones; and protein degradation can be avoided with subcellular targeting, protease mutants and co‐expressing protease inhibitors. We summarise the reported increased yields for various recombinant proteins achieved with these approaches and highlight remaining challenges to further improve the efficiency of this versatile protein expression platform.

Transient expression by infiltrating *Nicotiana benthamiana* leaves with *Agrobacterium tumefaciens* carrying genes of interest (agroinfiltration) is routinely used in plant science and molecular pharming. By transiently expressing genes of interest, one can investigate their roles in various biological processes without the need for stable transformation. Therefore, agroinfiltration facilitates the rapid assessment of gene function in a high‐throughput manner, enabling more efficient functional genomic studies. The ability of agroinfiltrated *N. benthamiana* to rapidly produce large amounts of foreign proteins also makes it an attractive platform for molecular pharming. This method offers several advantages over other expression systems, such as mammalian cell culture and microbial fermentation. The plant‐based transient expression typically has lower production costs and reduced risk of human pathogen contamination, is scalable, and has the potential for complex protein modifications including glycosylation. However, transient expression in *N. benthamiana* by agroinfiltration is still a challenge for many proteins, including antibodies and transmembrane glycoproteins, and can be further optimised. Here, we highlight four main processes that one needs to avoid to improve transient protein expression (Fig. 1).

**Fig. 1:**
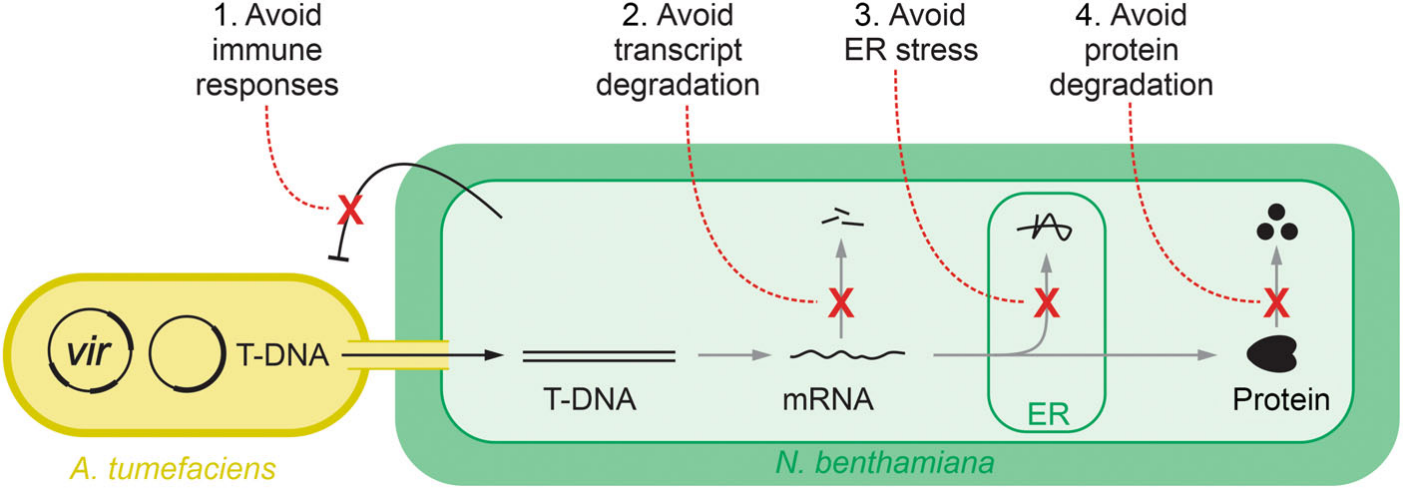
Four phenomena to avoid to improve transient protein expression. Transient expression by infiltrating leaves of *Nicotiana benthamiana* with *Agrobacterium tumefaciens* is mediated by the transfer DNA (T‐DNA) that is injected by the bacterium into the plant cell through the type‐IV secretion system. Transient expression can be improved by avoiding immune responses; avoiding transcript degradation (silencing); avoiding endoplasmic reticulum (ER) stress caused by protein accumulation in the endoplasmic reticulum; and by avoiding protein degradation by plant proteases.

## Avoiding immune responses

Agroinfiltrated zones of *N. benthamiana* leaves normally show only weak chlorosis. However, various studies on the transcriptome, proteome, and metabolome revealed that extensive cellular reprogramming is taking place as cell homeostasis is deprioritised whilst immune responses increase (Table 1). Nearly 25% of the transcripts show differential abundance after agroinfiltration (Grosse‐Holz *et al*., 2018a). Upregulated genes are those involved in pathogen perception, immune signalling, protein folding, oxidative stress, and lignification (Grosse‐Holz *et al*., 2018a; Hamel *et al*., 2023b). Downregulated genes include genes for photosynthesis and housekeeping proteins, consistent with the chlorotic response. Also, SWEET family sugar efflux transporters are downregulated (Grosse‐Holz *et al*., 2018a; Hamel *et al*., 2023b), perhaps to reduce the viability of microbes in the apoplast (Chen, 2014). Within the extracellular proteome, 70% of the proteins increase in abundance upon agroinfiltration, and several without significant change in transcript abundance, suggesting post‐transcriptional regulation (Grosse‐Holz *et al*., 2018a,b). This includes genes encoding pathogenesis‐related proteins, cell wall remodelling proteins, molecular chaperones, and several lipases and esterases (Grosse‐Holz *et al*., 2018a; Hamel *et al*., 2023b). Metabolomic changes include increased concentrations of phytol and α‐tocopherol, consistent with chlorophyll degradation, and high levels of chlorogenic acid derivates, consistent with lignification (Drapal *et al*., 2021). Collectively, these transcriptome, proteome, and metabolome changes are outputs of a basal immune response, similar to pattern‐triggered immunity induced by microbe‐associated molecular patterns (Zhang & Zhou, 2010). Consequently, various strategies can be taken to avoid or suppress immune responses upon agroinfiltration of *N. benthamiana* to improve transformation efficiencies. Avoidance of immune responses can be achieved by depleting immune receptors that recognise Agrobacterium in *N. benthamiana*. *Nb*CORE, for instance, is an immune receptor that recognises cold shock proteins of Agrobacterium but is expressed only in older *N. benthamiana* plants (Wang *et al*., 2016). Depletion of *Nb*CORE with virus‐induced gene silencing caused an eightfold higher transient green fluorescent protein (GFP) expression in older plants (Dodds *et al*., 2023). Meanwhile, suppression of immune responses has been achieved with bacterial type‐III effector AvrPto, which inhibits immune‐related kinases (Xing *et al*., 2007). Agrobacterium expressing AvrPto and the type‐III secretion system have increased transformation efficiencies in various plants, including *N. benthamiana* (Raman *et al*., 2022). These, and other, approaches to avoid and suppress immune response can significantly increase transient expression efficiencies.

**Table 1:** Differential gene expression in *Nicotiana benthamiana* upon agroinfiltration.

Function	Transcriptomics	Proteomics	Metabolomics	References
Photosynthesis	↓	↓	↓	Grosse‐Holz *et al*. (2018a); Drapal *et al*. (2021); Hamel *et al*. (2023b)
Cell wall remodelling (mainly lignification)	↑	↑	↑	Grosse‐Holz *et al*. (2018a); Drapal *et al*. (2021); Hamel *et al*. (2023b)
Sugar depletion	↑	↑	–	Grosse‐Holz *et al*. (2018a);Drapal *et al*. (2021); Hamel *et al*. (2023b)
ROS generation	↑	↑	↑	Grosse‐Holz *et al*. (2018a); Hamel *et al*. (2023b)
Immune perception and signalling	↑	↑	n/a	Grosse‐Holz *et al*. (2018a); Hamel *et al*. (2023b)
Proteases and inhibitors	↑	↑	n/a	Grosse‐Holz *et al*. (2018a); Hamel *et al*. (2023b)
Lipases and esterases	↑	↑	n/a	Grosse‐Holz *et al*. (2018a); Hamel *et al*. (2023b)
Salicylic acid signalling and SAR	↑	↑	n/a	Grosse‐Holz *et al*. (2018a); Hamel *et al*. (2023b)
Chaperones and UPR‐related	↑	↑	n/a	Grosse‐Holz *et al*. (2018a); Hamel *et al*. (2023a,b)

↓, significant decrease in abundance; ↑, significant increase in abundance; –, no significant change; n/a, not assessed; UPR, unfolded protein response.

## Avoiding transcript degradation

Transcripts encoded by transfer DNA (T‐DNA) often become unstable through post‐transcriptional gene silencing (PTGS), which usually starts on the third day after agroinfiltration. PTGS is triggered by low levels of antisense RNA generated by random T‐DNA insertion and/or RNA‐dependent RNA polymerase, which results in double‐stranded RNA (dsRNA). dsRNA is a substrate for Dicer to generate small interfering RNAs (siRNAs) that target the degradation of homologous mRNAs. To overcome this, silencing inhibitor P19 of tomato bushy stunt virus is often co‐expressed to suppress PTGS by sequestering siRNAs (Lombardi *et al*., 2009). But also, various knockout lines with reduced PTGS machinery have been generated. The removal of dicer‐like proteins 2 and 4 in the *dcl2dcl4* double mutant of *N. benthamiana* resulted in higher transient expression levels (Matsuo & Matsumura, 2017; Matsuo, 2022). Likewise, the removal of RNA‐dependent RNA polymerase 6 (*rdr6*) resulted in higher transient expression levels of GFP than the wild‐type (WT) plants (Matsuo & Atsumi, 2019). However, in a combinatorial study, the *dcl2dcl4* plants support higher amounts of transiently expressed GFP and human fibroblast growth factor‐1 than WT and *rdr6* plants (Matsuo, 2022). Likewise, other approaches to increase mRNA stability also improve transient expression. Different plant‐derived untranslated regions (UTRs) can increase mRNA stability, even in the presence of P19 (Garabagi *et al*., 2012). Moreover, the hypertranslatable (HT) vector system incorporates virus‐derived elements to boost transcription and translation, by increasing gene copy number and suppressing PTGS simultaneously (Sainsbury *et al*., 2009; Peyret & Lomonossoff, 2013).

## Avoiding endoplasmic reticulum stress

Transient protein expression of proteins that are targeted to the secretory pathway can lead to endoplasmic reticulum (ER) stress due to the unfolded protein response. In plants, the ER quality control system promotes protein folding and processing of misfolded proteins (Strasser, 2018). Essential to quality control is the presence of folding factors and chaperones in the ER lumen to assist polypeptides in their correct folding. Chaperones include binding protein (BiP), HSP90 family proteins, calnexin, calreticulin, protein disulphide isomerases (PDIs), and peptidyl‐prolyl isomerases (Gupta & Tuteja, 2011). Upregulation of chaperones is an important ER‐stress response, and this often occurs in *N. benthamiana* upon agroinfiltration, especially when expressing large amounts of secreted proteins from other organisms (Ye *et al*., 2011; Margolin *et al*., 2020; Hamel *et al*., 2023a). ER stress also occurs upon the expression of high levels of membrane proteins, such as viral glycoproteins. For instance, proteomic analysis has confirmed the increased abundance of PDIs, CRT, BiP, and ER‐associated degradation components upon transient expression of a viral glycoprotein and an IgG antibody that trigger ER stress (Hamel *et al*., 2023a,b). Interestingly, different recombinant proteins may require specific chaperones for folding. Different IgG antibodies, for instance, either accumulate highly without triggering ER stress or accumulate poorly, associated with ER stress (Hamel *et al*., 2023a). To reduce ER stress upon agroinfiltration, molecular chaperones have been co‐expressed alongside the target product. Human proteins are thought to be better folded by human chaperones than by plant chaperones given the divergence of the latter. Indeed, co‐expression with human calreticulin caused a 13‐fold increase in the transient expression of HIV glycoprotein gp140, whilst avoiding the induction of ER stress marker genes (Margolin *et al*., 2020). Likewise, co‐expression with human calreticulin caused a threefold increase in the accumulation of the S protein ectodomain (Margolin *et al*., 2020; Song *et al*., 2022). The emerging message is that different recombinant proteins might require co‐expression with specific chaperones to alleviate ER stress and increase protein folding and accumulation.

## Avoiding proteolysis

Proteolysis is a huge obstacle in transient expression. Many recombinant proteins in agroinfiltrated leaves accumulate initially and then disappear at later timepoints, and sometimes accumulate as shorter fragments, which indicates their degradation by plant proteases. Degradation has been observed for various transmembrane glycoproteins and IgG antibodies and has been studied mostly for specific IgG antibodies. The *N. benthamiana* genome encodes for *c*. 1200 putative proteases but not all of these proteases degrade recombinant proteins, as they are organelle‐specific, and many are not expressed or are inactive in agroinfiltrated leaves (Jutras *et al*., 2020). Most relevant for secreted recombinant proteins are probably papain‐like Cys proteases (PLCPs), subtilisins, and pepsin‐like Asp proteases that are abundant and active in the apoplast (Niemer *et al*., 2014; Deveuve *et al*., 2020; Puchol Tarazona *et al*., 2021). There are three main strategies taken to reduce proteolysis (Table 2). First, co‐expression with protease inhibitors has increased yields of recombinant proteins. Co‐expression with *Sl*CYS8, for instance, caused a threefold increase in the accumulation of full‐length IgG antibody H10 (Jutras *et al*., 2016). Likewise, co‐expression with *Nb*PR4, *Nb*Pot1, or *Hs*TIMP has increased the accumulation of IgG antibody VRC01, glycohormone erythropoietin, and α‐galactosidase (Grosse‐Holz *et al*., 2018b). Second, potentially harmful proteases can be depleted by silencing or genome editing. Transient depletion of vacuolar processing enzymes and PLCP *Nb*CysP6, for example reduced degradation of anti‐HIV antibody CAP256 (Singh *et al*., 2022). Finally, proteolysis can be prevented by targeting the protein to different subcellular locations. For instance, targeting proteins to the vacuole or retaining them in the ER has increased yields of transiently expressed 14D9 antibody by 10‐ to 15‐fold (Ocampo *et al*., 2016). Targeting recombinant proteins to protein bodies might be another way to avoid proteolysis (Schwestka *et al*., 2023). However, sensitivity to proteolysis very much depends on the recombinant protein itself, and it seems unlikely that a single strategy will avoid proteolysis for all recombinant proteins.

**Table 2:** Tackling proteolysis of recombinant proteins in agroinfiltrated *Nicotiana benthamiana*.

Approach	Description	Protein and accumulation^1^	Component	References
Protease inhibitor	Co‐expression with protease inhibitors	C5‐1 IgG antibody (LC; 70–80%)	*Sl*CDI; *Sl*CYS9	Goulet *et al*. (2012)
		C5‐1 IgG antibody (HC; 85%)	*Sl*CDI	Goulet *et al*. (2012)
		C5‐1 IgG antibody (40%)	*Sl*CYS8	Robert *et al*. (2013)
		H10 IgG antibody (HC; 7.5‐fold)	*Sl*CYS8	Jutras *et al*. (2016)
		α‐Galactosidase (4–14%) Erythropoietin (16‐ to 27‐fold) VRC01 IgG antibody (2‐ to 10‐fold)	*Nb*PR4, *Nb*Pot1 & *Hs*TIMP	Grosse‐Holz *et al*. (2018b)
Protease knockdown/out	RNAi and gene editing	CAP256 IgG antibody (−)	*Nb*VPE‐1a, *Nb*VPE‐1b, and *Nb*CysP6	Singh *et al*. (2022)
Recombinant protein compartmentation	Retention to ER or storage in protein bodies; vacuolar targeting	Fungal xylanase xyn11A (10‐fold)	HFBI	Saberianfar *et al*. (2015)
		Erythropoietin (twofold)	ELP	Saberianfar *et al*. (2015)
		Erythropoietin (twofold)	HFBI	Saberianfar *et al*. (2015)
		Interleucin‐10 (threefold)	HFBI	Saberianfar *et al*. (2015)
		14D9 IgG antibody (10‐ to 15‐fold)	ER; vacuole	Ocampo *et al*. (2016)
pH modulation of the plant secretory pathway	Co‐expression with proton channels	α1‐antichymotrypsin (fivefold)	Influenza M2 ion channel	Jutras *et al*. (2015, 2018)
		H3 influenza A (increase; ns)		
		HA influenza B (increase; ns)		

ELP, elastin‐like polypeptide; H, hemagglutinin; HFBI, hydrophobin; VPE, vacuolar processing enzymes.

^1^ Approximate accumulation according to authors' statements, − indicates no significant change, n/s indicates not specified.

## Future prospects

Although agroinfiltration is already a great platform for protein expression, there are still numerous opportunities ahead of us to further improve this platform. Besides the four discussed areas, there is even more to gain from optimising co‐expression, engineering post‐translational modifications, targeting other subcellular locations, improving the protein extraction process, producing metabolites, and even optimising plant growth and agroinfiltration conditions. Much of these activities require ingenuity and the development and application of new scientific insights. There is an exciting time ahead of us.

## Competing interests

None declared.

## Author contributions

KB, ECW and RALH conceived the topic and wrote the manuscript together. KB and ECW contributed equally to this work.
